# Direct bladder hernia after indirect hernia repair in extremely low birth weight babies: two case reports and a review of the literature

**DOI:** 10.1186/s13256-016-1171-5

**Published:** 2017-01-16

**Authors:** R. B. Tröbs, B. Yilmaz, C. Roll, M. Alrefai

**Affiliations:** 1 Department of Pediatric Surgery, St. Mary’s Hospital Herne, St. Elisabeth Group, Ruhr-University of Bochum, Widumer Str. 8, 44627 Herne, Germany; 2 Clinic of Surgery and Pediatric Surgery, Friedrich-Ebert-Str. 13, 33699 Bielefeld, Germany; 3 Center of Perinatology, Department of Neonatology and Pediatric Intensive Care, Vest Children’s Hospital, University of Witten-Herdecke, Dr.-Friedrich-Steiner Str. 5, 45711 Datteln, Germany

**Keywords:** Bladder hernia, Pediatric hernia, Direct hernia, Hernia relapse, Case report

## Abstract

**Background:**

Inguinal hernia repair is the most common surgical procedure in babies. Despite a meticulous technique, relapses may occur. The occurrence of a direct bladder wall hernia in relapses has never before been reported in the literature.

**Case presentation:**

Here, we report two cases of direct bladder herniation: a white baby boy born after 25 weeks of gestation and a white baby boy born after 26 weeks of gestation. Both of the formerly extremely low birth weight babies were affected after open bilateral hernia repair. Recurrent hernias developed on the right side, and direct bladder herniation was identified intraoperatively. In one case, laparoscopy was applied to identify a supravesical type of hernia. Immaturity and a difficult postnatal course might have contributed to hernia relapse in these cases.

**Conclusions:**

Misinterpretation of bladder herniation might have disastrous consequences. Laparoscopy is a helpful tool in comparable cases.

## Background

The incidence of inguinal hernias is much higher among preterm babies than among term-born babies, children, and adults. Such hernias affect 30% of babies with birth weights (BWs) below 1000 g [1], and surgical repair in preterm babies can be challenging. The optimal timing for hernia repair in these delicate patients remains controversial. Early repair may prevent incarceration and is therefore recommended by many pediatric surgeons [2]. However, major concerns associated with early repair include cardiorespiratory instability during and after surgery and increasing evidence that anesthetic and sedative agents may have direct toxic effects on the developing brains of preterm babies even after these babies have reached postmature age. Therefore, surgery is often delayed until term-equivalent age, particularly for the most vulnerable preterm babies [3].

Relapses might occur as indirect or direct hernias [4]. Rare forms of inguinal hernias may occur [5], and the surgeon must be aware that the urinary bladder can be involved in indirect and direct hernias [6–16].

Here we report two cases of secondary bladder hernia in babies after the open repair of bilateral indirect inguinal hernias. To the best of our knowledge, the occurrence of a direct bladder wall hernia after primary inguinal hernia repair in babies has never before been reported in the literature.

## Case presentation

We retrospectively identified two cases involving bladder herniation between 2010 and 2014. During this 5-year observation period, 1100 inguinal hernia repairs were performed in the reporting pediatric surgical unit, with the vast majority of patients below 1 year of age. In both reported cases, we initially performed hernia repair under caudal anesthesia. Shortly afterward, the external ring was exposed with an incision of a few millimeters, and the hernia sac and cord structures were separated. After the sac was dissected, closure was performed with a purse string suture (Case 1) or transfixation (Case 2). In addition, this suture was used to anchor the stump of the sac within the inguinal channel (backward suture through an internal oblique muscle and an external oblique fascia and tied externally). Any excess sac was cut away. The inguinal canal was reconstructed by fixation of the internal oblique muscle to the inguinal ligament with one stitch, and the edges of the external oblique fascia were adapted to the inguinal ligament with two stitches.

### Case 1

**Fig. 1:**
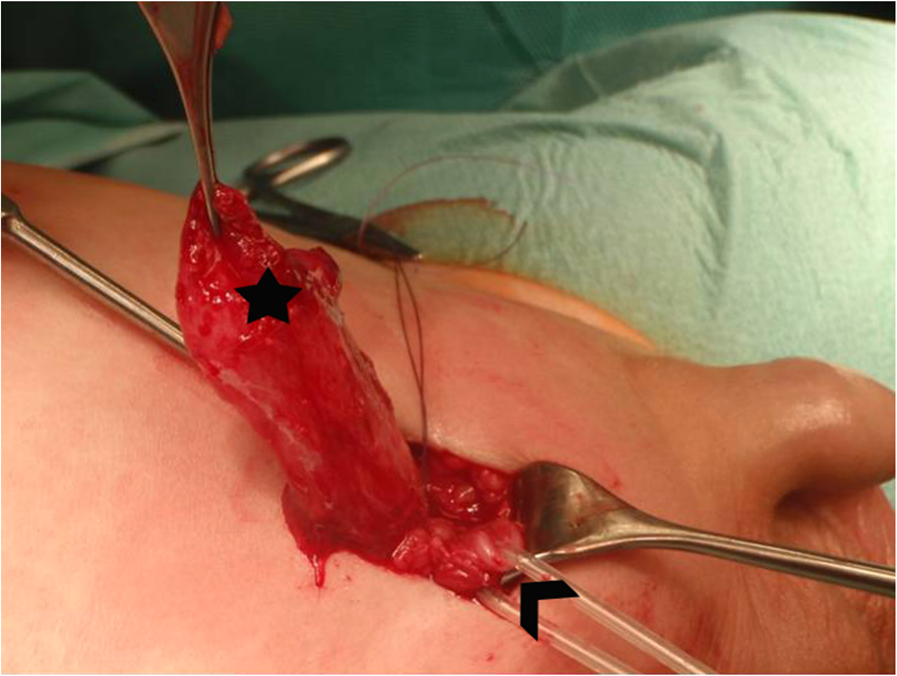
The protruded bladder (*star*) mimics a thickened hernia sac. The funiculus (*arrow head*) is separated by a loop

**Fig. 2:**
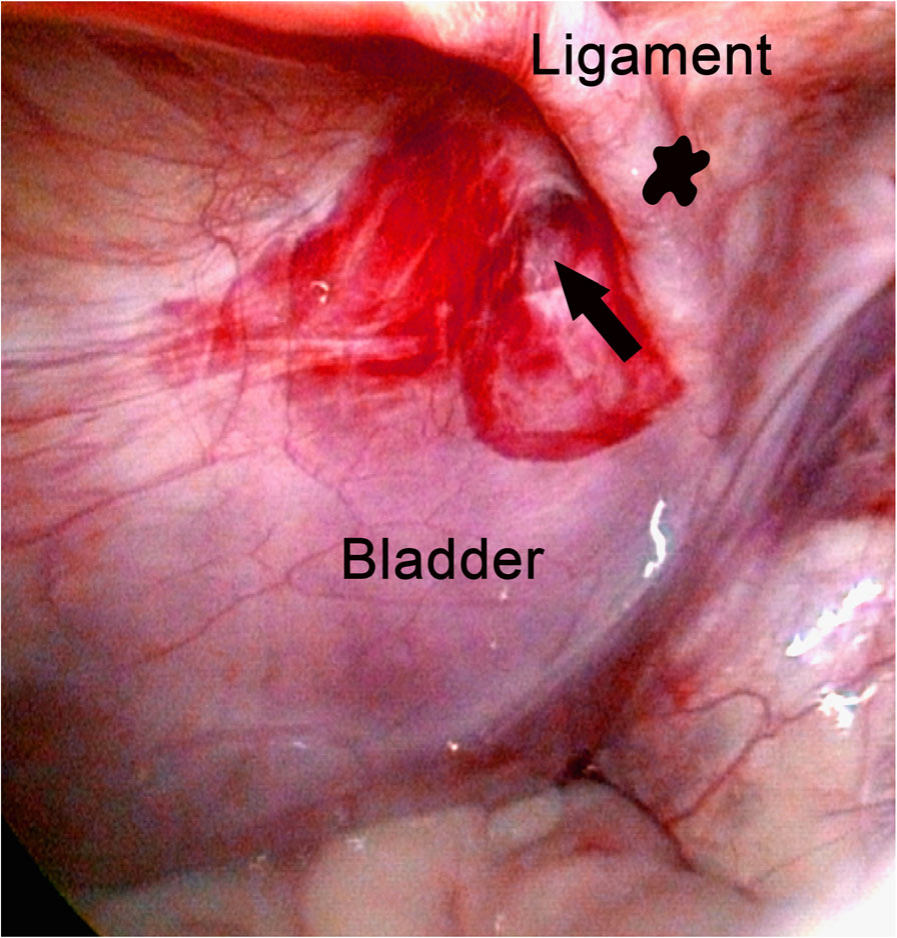
Laparoscopic view after bladder hernia repair. A peritoneal dimple above the bladder (*arrow*) and medially to the medial umbilical fold (*star*) indicates the former hernia site

A white baby boy was born after 25 weeks of gestation with a BW of 600 g. He had respiratory distress syndrome that was treated with surfactant in the delivery room and was on mechanical ventilation from birth, followed by continuous positive airway pressure (CPAP) for 2 months. At postnatal ultrasound, residuals of a first-degree prenatal intraventricular hemorrhage were obvious. He developed moderate bronchopulmonary dysplasia and presented with large scrotal bilateral inguinal hernias. At a prior surgery, no clinical signs of neurological impairment were observed by the neonatologist. Bilateral hernia repair was performed at the postnatal age of 4 months (a corrected age of 42 weeks) when his body weight was 4050 g. At the age of 11 months, recurrent inguinal bulging at the level of the scar of his right inguinal region occurred, and a unilateral relapse was evident. Both testes were descended and of adequate size. Laparoscopic surgery was begun, and a small dimple at the right internal inguinal ring was visible. However, no internal hernia ring could be identified. Because a hernia sac was clearly externally palpable, open exploration was used to continue the surgery. After the identification of the external aponeurosis, a thick-walled hernia sac was identified (Fig. 1). A 6 Ch transurethral catheter was inserted. The filling of his bladder with saline enabled the identification of his protruded bladder. After bladder repositioning, reconstruction of the transversalis fascia and the muscular borders was performed. Repeated laparoscopy at the end of the procedure revealed the anatomical constellation of a prior supravesical hernia (Fig. 2). No recurrence was observed over a 4-year follow-up period (information from the family’s pediatrician).

### Case 2

A white baby boy was born after 26 weeks of gestation with a BW of 506 g; he was small for gestational age (SGA). He had respiratory distress syndrome and was treated with mechanical ventilation followed by CPAP therapy. Bilateral scrotal hernias developed; at 3 months postnatal age (a corrected age of 40 weeks), bilateral open closure of these indirect hernias was performed. Because his right testis was in an inguinal high position, orchidopexy according to Shoemaker was required.

**Fig. 3:**
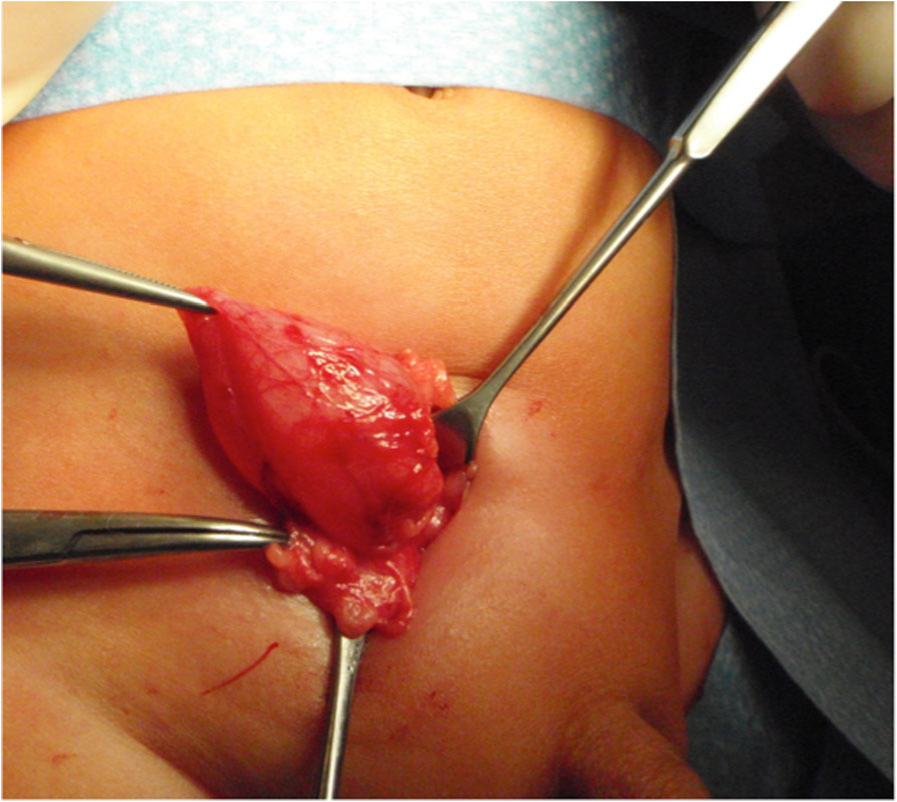
The protruded urinary bladder is found after incision of the former transverse inguinal incisional scar (second case). Preperitoneal fat is seen below the bladder

His postoperative course was complicated by fever, desaturations, and elevated C-reactive protein (10.3 mg/dL) due to septicemia caused by *Staphylococcus aureus.* No external signs of wound infection occurred. Two months later, recurrent groin swelling that spontaneously reduced was observed on his right side. An open inguinal revision was begun, and a direct bladder hernia separate from the funiculus was found (Fig. 3) in a more medial position. Protruded fat indicated the herniation of his anterior bladder through the prevesical space. After bladder repositioning, two-layer closure of the hernia ring was performed. Revision of the funiculus revealed no further pathology. During 4 months of follow-up, no recurrence was observed, and both testes were in a scrotal position.

Table 1 provides a short summary of both cases.

**Table 1:** Summary of the two reported cases

Patient	Gestational age	Birth weight	Postnatal (corrected) age	Weight at surgery	Primary hernia	Postoperative course	Time to relapse	Side of bladder hernia
1	25 wk.	600 g	4 months (42 wk.)	4050 g	Large scrotal hernia on the left, smaller scrotal hernia on the right	Uneventful	7 months	Right
2	26 wk.	506 g (SGA)	3 months (40 wk.)	2520 g	Large bilateral scrotal hernias	Sepsis (*Staphylococcus aureus*)	2 months	Right

*SGA* small for gestational age, *wk.* weeks

## Discussion

Cadaveric and radiographic studies have indicated that transitory extraperitoneal herniation of the bladder, a phenomenon known as “bladder ears,” may occur in significant numbers of babies, children, and even adults [13]. According to these observations, the bladder may herniate into the internal ring, and the bladder’s grade of protrusion depends on bladder filling.

Among adults, the incidence of bladder involvement in low abdominal hernias reportedly ranges from 1 to 10% [6]. Direct and indirect hernias are involved with equal frequency, with hernias predominantly occurring on the right side [6]. 

The rate of bladder involvement during indirect inguinal hernia repair is not well known. Shaw and Santulli used a flap technique to repair sliding hernias with involvement of the urinary bladder in 27 female patients [1]. Several additional case series describe bladder involvement during pediatric hernia repair. Six cases with recognized bladder involvement and one case involving unrecognized bladder injury were reported by Colodny [2]. In contrast, in a large single-surgeon series of 6361 cases, only two patients (0.03%) had sliding bladder hernias [9].

Reports on pediatric bladder hernias and certain near-catastrophic iatrogenic urinary bladder injuries are collected in Table 2. Eight out of the nine presented cases involved male patients. Bladder hernias affected the left side in three cases and the right side in two cases. In three cases, the affected side was not reported, and in one extraordinary case, a median protrusion occurred. No patients in the presented series had previously undergone surgery for inguinal hernia repair. Injuries of the bladder occurred due to either the prolapse of the bladder into the indirect hernia sac or a misplaced approach medial to the inguinal canal.

**Table 2:** Reports on pediatric bladder injuries at the time of inguinal hernia repair

Reference	Age at surgery	Sex	Side	Type of hernia	Surgical finding	Assumed mechanism
[1] Shaw and Santulli 1967	3.5 mo.	M	Not given	Inguinal hernia, bilateral	Partial excision of the bladder	Either paraperitoneal or intraperitoneal bladder herniation into the indirect hernia sac
[1] Shaw and Santulli 1967	9 mo.	M	Not given	Not given	Not given	
[2] Colodny 1974	3 mo.	M	Left	Inguinal hernia, incarcerated	Purse string on the left side of the bladder	Bladder injured with the closure of the hernia sac
[6] Bell and Witherington 1980	1 yr.	M	Midline	Infraumbilical fascial defect	Fascial closure without problems	Prematurity, penis malformation
[3] Redmann *et al*. 1985	4.5 wk.	M	Right	Inguinal hernia, bilateral	Subtotal bladder resection, dissection of the funiculus	Bladder incorporated into the indirect hernia sac
[3] Redmann *et al*. 1985	5 wk.	M	Left	Inguinal hernia, bilateral	Near-total bladder resection, dissection of both ureters	
[4] Chung and Yu 1999	18 mo.	M	Right	Inguinal hernia	Near-total bladder resection	Bladder incorporated into the indirect hernia sac
[14] Koot *et al*. 1998	3 mo.	F	Left	Inguinal hernia	Bladder open at the top, persistent indirect hernia on the left	Prematurity, overly medial incision and dissection of the transversalis fascia
[5] Bakal *et al*. 2015	Infant	M	Not given	Inguinal hernia	Intraperitoneal and extraperitoneal injury	Incision on the medial site of inguinal canal

*F* female, *M* male, *mo.* months, *wk.* weeks, *yr*. year

In contrast with the observations above, bladder herniation occurred in our patients as a relapse after the primary repair of indirect inguinal hernias. In both cases, the bladder wall was prolapsed directly through an opening in the prevesical abdominal wall. The medial margin of this opening was lined by the lateral border of the rectus sheath (Hesselbach’s triangle) [17].

In general, hernia recurrence rates of 0.8 to 3.8% have been reported following open hernia repairs in children [11]. An increased incidence of direct herniation after the repair of congenital indirect hernias is a well-known phenomenon. In a previous series, 20 out of 62 children had direct hernias [11]. In another series of over 1600 inguinal hernias, six patients presented with nine recurrent hernias [18].

Several factors that may predispose patients to indirect inguinal hernia recurrence have been reported. Hernia recurrence in otherwise healthy children may be caused by inadequate surgical technique (failure to ligate the sac sufficiently high and inadvertent tearing of the sac) [18]; injury to the floor of the inguinal canal due to operative trauma; the inherent weakness or friability of tissues; and postoperative wound infection or hematoma [11]. In the two presented cases, the primary surgery and the repair of the relapse were performed by the first author (RBT). The applied technique was a modified “classical” hernia repair, as described in detail by Ladd and Gross [8]. For both babies, during relapse surgery, surgical exploration through the previously used inguinal access revealed a right extraperitoneal bladder hernia. This predilection for the right side may be supported by the fact that 59% of indirect hernias occur on the right side [9]. 

Laparoscopy was an important tool for specifically localizing the type of relapsed hernia in Case 1; however, laparoscopy was not used in our second case. In both cases, direct bladder herniation occurred extraperitoneally in the form of a direct hernia.

In both reported cases, the family history was unremarkable, and there were no clinical signs of relevant neurological impairment. Prematurity and multimorbidity might have contributed to the development of secondary bladder herniation. Increased intra-abdominal pressure due to CPAP ventilation and repeated squeezing of the immature intestine are additional predisposing factors. In our cases, the role of the primary surgical technique remains debatable. The narrowing of the enlarged inguinal canal by placing a suture at the margin of the internus muscle and the aponeurosis of the obliquus externus might have contributed to weakening the medial portion of the inferior abdominal wall.

## Conclusions

The surgeon should take into account that bladder herniation might occur in babies with inguinal hernia relapse. Laparoscopy allows identification of the underlying type of hernia. Bladder injury has to be avoided.

## Abbreviations

BW: Birth weight
CPAP: Continuous positive airway pressure
SGA: Small for gestational age
